# Catechin Hydrate Augments the Antibacterial Action of Selected Antibiotics against *Staphylococcus aureus* Clinical Strains

**DOI:** 10.3390/molecules21020244

**Published:** 2016-02-20

**Authors:** Maria Miklasińska, Małgorzata Kępa, Robert D. Wojtyczka, Danuta Idzik, Arkadiusz Dziedzic, Tomasz J. Wąsik

**Affiliations:** 1 Department of Microbiology and Virology, School of Pharmacy and Division of Laboratory Medicine in Sosnowiec, Medical University of Silesia, ul. Jagiellońska 4, 41-200 Sosnowiec, Poland; maria.miklasinska@o2.pl (M.M.); mkepa@sum.edu.pl (M.K.); rwojtyczka@sum.edu.pl (R.D.W.); didzik@sum.edu.pl (D.I.); 2 Department of Conservative Dentistry with Endodontics, Medical University of Silesia, Katowice, Pl. Akademicki 17, 41-902 Bytom, Poland; adziedzic@sum.edu.pl

**Keywords:** catechin hydrate, antibacterial activity, *Staphylococcus aureus*

## Abstract

Synergistic effects between commonly used antibiotics and natural substances may be an alternative to conventional antibacterial therapies. The objective of the presented study was to assess the *in vitro* antibacterial activity of catechin hydrate (CH) and evaluate the interactions of CH with selected antibiotics using *Staphylococcus aureus* clinical and reference strains. CH displayed diverse activity towards examined *S. aureus* strains, with minimal inhibitory concentrations (MICs) ranging from 256 to 2048 µg/mL. The interaction between CH and antibiotics was assessed by an E-test. The most significant synergistic effects were noticed for CH in combination with clindamycin and erythromycin. For cefoxitin and vancomycin a decrease of MIC values in the presence of CH was also observed, but it did not reach statistical significance. The obtained results demonstrate that CH shows antimicrobial activity against *Staphylococcus aureus* clinical strains. What is more, we proved a synergistic effect of CH with erythromycin and clindamycin.

## 1. Introduction

The research concept of the study presented herein resulted from the worldwide escalation of clinical infections caused by multi-drug resistant microorganisms and the spread of resistance among bacteria to commonly used antibiotics. At present one of the main public health problems is associated with the infections caused by multi-drug resistant bacteria, so it is clear that the search for new antibacterial agents is absolutely necessary [1]. Methicillin resistant *Staphylococcus aureus* (MRSA) strains, which are resistant to nearly all current anti-staphylococcal drugs, pose a particular threat, especially for hospitalized patients with decreased immunity [2,3]. The spread of the drug resistance among *S. aureus* strains has stimulated the search for the new strategies for the treatment of multi-drug resistance infections. The best way to fight such infections would be to develop a new class of potent antibacterial agents, yet such an approach is extremely expensive and time consuming. What is more, the probability of success is rather disputable. Another way to overcome bacterial resistance is to find compounds which can improve the antibacterial effect of commonly used antibiotics [3,4]. Many studies have demonstrated that some natural flavonoids have antimicrobial properties against a broad spectrum of microorganisms and may be an alternative to conventional therapy [5,6,7,8,9,10,11,12,13,14,15], therefore we undertook research to discover new natural compounds with antibacterial properties which can contribute to the fight against multi-drug resistant bacteria.

Catechin is a flavonoid (flavan-3-ol) found, for example, in tea, wine, some fruits, vegetables and chocolate [16]. It has been proved that catechin has radical scavenging [17,18,19] metal-chelating [20,21] and antiproliferative effects [22,23]. It also has been demonstrated that (+)-catechin has the ability to inhibit HIV-1 reverse transcriptase *in vitro* [12,24]. Many studies have indicated that flavan-3-ols also possess antibacterial activity. The antimicrobial properties of tea have been known for over a century and its bacteriostatic and bactericidal effects have been assessed [25]. Catechins possess the ability to bind to artificial lipid bilayers [26,27,28,29,30]. It has been shown that the catechins (−)-epicatechin gallate and (−)-epigallocatechin gallate can sensitize MRSA strains to β-lactam antibiotics, including methicillin [25,31,32,33,34]. It has been also reported that epicatechin gallate and epigallocatechin gallate acted as a *norA* gene suppressors [35] and decreased β-lactam MICs to the antibiotic breakpoint, thus increasing the antimicrobial activity of those antibiotics [3,31,33,36,37]. Kubo *et al.* proved the direct antibacterial activity of alkyl gallates [38].

The majority of conducted studies on the antimicrobial activity of catechins focused on epicatechin and gallates of epicatechin [25,32,33,37,39,40] as the main representatives of flavonoids but reports on antimicrobial properties of catechin are scarce. Research on the antibacterial activity of (+)-catechin has demonstrated that it has an ability to reduce the biosynthesis of the virulence factors which control a quorum-sensing system in *Pseudomonas aeruginosa* [41]. It has also been shown that polymerized catechin suppresses the activity of *Staphylococcus aureus* α-toxin and that CH is an effective urease inhibitor in *Staphylococcus saprophyticus* strains [42,43]. The above observations prompted us to investigate *in vitro* antimicrobial activity of CH against *S. aureus* clinical strains.

## 2. Results

### 2.1. Identification of Examined Strains

All the examined strains were classified as members of *Staphylococcus aureus* species. The profiles of resistance to methicillin and MLS_B_ antibiotics obtained for the analyzed strains are presented in Table 1.

### 2.2. Antimicrobial Activity of the Catechin Hydrate (CH)

CH at the concentrations used in this study inhibited the growth of all tested staphylococci strains. The MICs obtained for CH ranged from 256 for the most susceptible strains, to 2048 µg/mL for the most resistant strains, with a median values of 1024 µg/mL (Table 1). The antimicrobial activity of CH against the examined *S. aureus* strains was identical for 16 strains, with MICs at 1024 µg/mL. CH showed the strongest antibacterial activity against *Staphylococcus aureus* ATCC 25923, *S. aureus* ATCC 6538 reference strains and *S. aureus* 13 clinical strain with MICs of 256 µg/mL, while against *S. aureus* 1, 3, 6, 17 strains CH demonstrated weaker antibacterial activity what resulted in much higher MIC values: 2048 µg/mL (Table 1). Comparison of MRSA and MSSA strains demonstrated that in both cases the MIC values ranged from 256 to 2048 µg/mL. A statistical analysis indicated that there were no significant differences between MIC values for MRSA and MSSA strains (*p* = 0.203), as well as for MLS_B_ negative, kMLS_B_ and iMLS_B_ strains (*p* = 0.632).

### 2.3. Combined in Vitro Effects of CH and Antibiotics

The antimicrobial activity of CH with erythromycin (E), clindamycin (DA), cefoxitin (FOX) and vancomycin (VA) is shown Table 2. Addition of one-fourth of the MIC of CH to the Mueller Hinton agar (MHA) increased sensitivity of the tested staphylococci to all examined antibiotics. In case of E (*p* = 0.009), and DA (*p* = 0.006) the results reached statistical significance. Decrease of MICs for VA-CH combination (*p* = 0.605) and FOX-CH (*p* = 0.064) combination was not significant.

The diminished MICs in the presence of CH ranged from 24 to even 99% (Table 2). The most susceptible strains to a synergistic effect of the CH and antibiotics were *S. aureus* 1, 4, 5 and 7 strains, which were more sensitive to all antibiotics after MHA supplementation with CH. The strongly augmented effect of CH in the presence of all antibiotics was also noted for *S. aureus* strain 8, except for VA, *S. aureus* strain 11 except for FOX and *S. aureus* strain 18 except for DA.

Some strains were found to be resistant to CH in combination with the tested antibiotics. No MIC changes were observed in *S. aureus* strains 10, 16 and 19. The level of resistance to E, DA and FOX was not affected by the MHA supplementation with CH for *S. aureus* strains 12, 13, 14, 16, 17, 19, 20. Furthermore, for some strains and some antibiotics after CH addition antagonistic interactions were noted. The increases of MICs ranged from 32% to even 197% (Table 2).

Statistical analysis excluded the significance of the differences between the changes of MIC values for MRSA and MSSA strains for E − *p* = 0.363; DA − *p* = 0.221, FOX – *p* = 0.082 and VA − *p* = 0.391. There were no significant differences between changes of MICs obtained for MLS_B_ negative *vs.* kMLS_B_ and iMLS_B_ staphylococci in case of E − *p* = 0.166; FOX − *p* = 0.621 and VA − *p* = 0.922, while for DA statistical analysis revealed significant differences (*p* = 0.038). Further analysis demonstrated that strains with kMLS_B_ phenotype of resistance showed lower susceptibility to the combination of CH with DA than MLS_B_ negative strains.

## 3. Discussion

It is well know that catechins have antimicrobial activity against both Gram-positive and Gram-negative bacteria. Catechins exert indirect antimicrobial activity by modifying the resistance level to antibiotics and by changing gene expression associated with the bacterial virulence. The majority of the research on the flavonoids antibacterial activity was focused on epicatechin and gallates of epicatechin alone or in the combination with antibacterial agents [25,27,32,33,39,40,45]. To our knowledge the present study is one of the first to explore the antimicrobial activity of CH.

Many studies have showed that catechins possess the capacity to reverse oxacillin resistance in *S. aureus* [26,32,36,37], which is also observed in epicatechin gallate. Stapleton *et al.* studied the antibacterial activity of catechins, including (+)-catechin on three *S. aureus* strains—BB568, EMRSA-15 and EMRSA-16—and demonstrated that MRSA strains were insensitive to (+)-catechin with MICs > 256 mg/L [31]. In our study the MIC values of CH obtained for MRSA reference and clinical strains ranged from 256 to 2048 µg/mL, with a median value of 1024 µg/mL. A study on the antimicrobial activity of catechins alone and in combination with oxacillin against MRSA strains was carried out by Stapleton *et al.* in 2004 on the strains described above [26]. The obtained MIC values were the same as the ones in the authors’ previous work, so to enhance the capacity of catechins to interact with cytoplasmic membrane and consequently to increase their antibacterial activity the acyl chains were incorporated into (+)-catechin. MICs obtained after addition of acyl chains were smaller and ranged from 16 to 256 mg/L [26].

Park *et al.* studied the antibacterial activity of 3-*O*-alkyl analogues of (+)-catechin against Gram-positive and Gram-negative bacteria and demonstrated that alkylation improved the activity of a parent compound and antibacterial properties increased in relations with the number of carbons in the alkyl chain [46]. Similarly, Holloway *et al.* in their study tried to increase antibacterial activity of catechin and found out that metal (II) ions such as copper (II) sulphate and iron (II) enhance antibacterial activity of (+)-catechin against *S. aureus* strains due to increased H_2_O_2_ production after metal ions supplementation [21]. Perhaps the incorporation of substituents to CH could also increase its antibacterial properties.

The studies on the combined action of catechin and epicatechin gallate with β-lactams against standard and clinical MRSA strains were carried out by Qin *et al.* [3]. Catechin alone did not increase the susceptibility of MRSA strains to β-lactam antibiotics with MICs > 1024 mg/L. In our study we showed that CH alone increased susceptibility of MRSA strains to the β-lactam antibiotic cefoxitin, but it did not prove to be statistically significant. However, Qin *et al.* reported that a catechin-epicatechin gallate combination increased MRSA strains susceptibility to β-lactam antibiotics and this effect was related to the catechin, but not to epicatechin gallate concentration. What is more, catechin showed higher activity than *cis* forms of non-galloylated catechins such as (−)-epicatechin or (−)-epigallocatechin in enhancing MRSA susceptibility to β-lactam antibiotics. Qin *et al.* indicated that catechin-epicatechin gallate combination enhances activity of such antibiotics as ampicillin, ampicillin/sulbactam, cefazolin, cefepime and imipenem/cilastatin which usually cannot be used in the treatment of MRSA infections because of bacterial resistance. The authors suggested that these antibiotics could be used in treatment when combined with catechin and epicatechin gallate. Moreover, they pointed out that analyzed flavonoid-antibiotic combinations did not show similar effects when combined with non-β-lactam antibiotics. The authors suggested that the enhancement of an antibacterial effect of β-lactam antibiotics by catechin-epicatechin combination might be related to the accumulation of antibiotics and inhibition of efflux pump gene expression. In our work we demonstrated that CH decreased the MIC values of both β-lactam (cefoxitin) and non-β-lactam (erythromycin, clindamycin and vancomycin) antibiotics and the diminished of MICs in the presence of CH ranged from 24% to even 99%. Our research proved that CH exerted an antimicrobial activity toward the examined *S. aureus* strains. What is more, our results suggest that the combined action of CH and selected antibiotics demonstrates a synergistic effect. The MICs of CH against the tested staphylococci ranged from 256 to 2048 µg/mL and the vast majority of examined strains had a MIC of 1024 µg/mL. The statistical analysis showed that varying sensitivity of the tested staphylococci to CH was not affected by a methicillin resistance profile or a phenotype of resistance to MLS_B_ antibiotics. The mechanism of CH effect on bacterial cells is still unknown and we can only suppose that the most likely cause of discrepancies of the obtained MICs is related to the intra-species diversity, so further studies are needed to investigate the mechanism of the CH effect on bacterial cells.

Our analysis of the influence of CH on the antibacterial properties of the selected antibiotics revealed a substantial reduction of the MICs for all antibiotics. The most noticeable synergistic effect was observed for CH in combination with erythromycin and clindamycin. Synergism between CH and vancomycin, CH and cefoxitin was also observed, but it did not prove to be statistically significant. The profile of resistance to methicillin did not influence MICs changes, but the MIC changes of clindamycin following the CH addition were affected by the profile of resistance to MLS_B_ antibiotics. Strains resistant to clindamycin, erythromycin and cefoxitin (*S. aureus* ATCC 4330*, S. aureus* 12, 13, 14, 16, 17, 19, 20) were insensitive to CH-E, CH-DA and CH-FOX combinations and statistical analysis proved that strains with a constitutive phenotype of resistance were less susceptible to CH-clindamycin combination than staphylococci without MLS_B_ phenotype, what indicates that CH is not effective against kMLS_B_ strains.

Since CH showed the same action against sensitive and multi drug-resistant *S. aureus* strains it is therefore possible that the CH can be used in the treatment of both multi-drug resistant and drug susceptible bacterial infections. In this context, it is worth noting that the antibacterial activity of catechin can be improved by chemical modifications, as previous studies have showed [21,26]. The synergy between CH and antibiotics indicates the potential application of compound combinations as an efficient, novel therapeutic tool for treatment of multi-drug resistance infections.

## 4. Materials and Methods

### 4.1. Bacterial Strains and CH

The antimicrobial activity of CH was assessed against 20 *S. aureus* strains isolated from clinical wound samples, and three reference strains of *S. aureus*: ATCC 25923, *S. aureus* ATCC 43300 and *S. aureus* ATCC 6538. To ensure the homogeneity of the examined samples all tested strains were collected from surgical wounds. All bacterial strains were stored for further analyses in Soy Broth medium with 20% of glycerol at −80 °C. The CH used in this study was obtained from Sigma Chemical Co. (St. Louis, MO, USA) and dissolved in DMSO immediately prior to use.

### 4.2. Identification of Examined Strains

All the examined isolates were identified by the conventional methods including colony morphology, hemolysis, catalase and coagulase tests and the anaerobic fermentation of mannitol. Catalase and coagulase positive strains where identified by the API STAPH test (bioMerieux, Marcy-l’Étoile, France) according to the manufacturer’s instruction. The PCR-RFLP technique was also performed. For PCR-RFLP analysis, bacterial genomic DNA was extracted with the GeneMATRIX Tissue & Bacterial DNA Purification KIT (EuRx Ltd., Gdańsk, Poland) according to the manufacturer’s recommendations with the modification described by Shah *et al.* [47]. The *dnaJ* primers SA-(F) 5′-GCC AAA AGA GAC TATTAT GA-3′ and SA-(R) 5′-ATT GTT TAC CTG TTT GTG TAC C-3′ were used to amplify the *dnaJ* gene fragment. The PCR amplification was performed using 10× PCR RED master mix kit (BLIRT SA, Gdańsk, Poland) in a MJ Mini Personal Thermal Cycler (Bio-Rad, Hercules, CA, USA). PCR products were visualized under UV light following the electrophoretic separation in a 1.5% agarose gel (Promega, Madison, WI, USA) with ethidium bromide (Promega). To identify staphylococci strains, the PCR products were treated with 10U of restriction enzymes *Xap*I and *Bsp*143I (Fermentas, Vilnius, Lithuania). The obtained fragments were separated by electrophoresis in a 2% agarose gel with ethidium bromide (Promega), visualized under the UV light and their size was checked against 1 Kb HypeLadderIV (BLIRT SA, Gdańsk, Poland) molecular weight marker.

### 4.3. Determination of Methicillin Resistance Profiles

Disk diffusion method with a 30 µg disc of cefoxitin (EMAPOL, Gdańsk, Poland) and Mueller-Hinton Agar (MHA-BTL, Łódź, Poland) was used to determine resistance phenotype to methicillin. MRSA strains were detected using a breakpoint of ≤22 mm zone diameter size for cefoxitin disk.

The *mecA* gene detection was performed by PCR amplification according to the method described by Murakami *et al.* [48]. The following *mecA* primers (F) (5′-AAA ATC GAT GGT AAA GGT TGG C-3′) and (R) (5′-AGT TCT GCA GTA CCG GAT TTG C-3′) were used. The PCR reaction was carried out using 10× PCR RED master mix kit (BLIRT SA) in a MJ Mini Personal Thermal Cycler (Bio-Rad, Hercules, CA, USA). The amplicons were detected under UV light after electrophoresis in 1.5% agarose gel with ethidium bromide (Promega).

### 4.4. Disk Diffusion Method

The antibacterial susceptibility of examined strains to MLS_B_ antibiotics was examined by a disk-diffusion method and interpreted according to the EUCAST guidelines [44] with antibiotic discs (EMAPOL) with 15 clindamycin μg (DA), 2 μg erythromycin (E) and Mueller-Hinton Agar (MHA-BTL). The distance between the edges of disks was 15–16 mm, according to EUCAST recommendation. The staphylococci were classified as resistant or sensitive based on the zone diameter size and shape.

### 4.5. Microdilution Method

MIC is the lowest concentration of an antimicrobial that will inhibit the visible growth of a microorganism. MICs indicate and confirm the resistance of microorganisms to an antimicrobial agent and also to determine the potency of new antimicrobial agents [49]. In the presented study MICs were defined as the lowest CH concentration that yielded no visible growth after 24 h of incubation [50,51]The MICs of CH were determined by a microtiter broth dilution method. The growth inhibition assays were performed in sterile 96-well plates (FL Medical, Torreglia, Italy) in a final volume of 200 µL [50,51]. The cell concentrations were estimated from the optical densities at 600 nm wavelength with the formula CFU/mL = *A*_600_ (3.8 × 10^8^), where CFU was the number of colony-forming units. One hundred microliters of mid-logarithmic-phase bacterial cultures (5 × 10^5^ CFU/mL) in TSB was added to 100 μL of sterile dilutions of CH (2, 4, 8, 16, 32, 64, 128, 256, 1024 and 2048 μg/mL). Wells containing TSB with bacterial inoculum only served as a bacterial growth control (GC). The additional controls included TSB alone (medium sterility control) and TSB with different concentrations of CH and bacterial inoculum. All samples were prepared in triplicates. Microplates were incubated at 37 °C for 24 h, and the bacterial cell growth was assessed by measuring the optical density of cultures at 600 nm wavelength with a Multiskan EX microplate reader (Thermo Electron Corp., Vantoa, Finland) [52,53].

### 4.6. The Synergistic Effect of CH and Antibiotics

All analyzed strains were examined for the susceptibility to antibiotics by the E-test, using MHA and commercially available MIC Test Strips (Liofilchem, Roseto Degli Abruzzi, Italy) containing antibiotic concentration gradient according to the EUCAST recommendations [44]. For E-test, 90 mm plates with the agar medium were inoculated by swabbing the agar with a swab soaked in a bacterial suspension of 1× 10^8^ cells/mL. MIC Test Strips containing concentration gradient of erythromycin (E), clindamycin (DA), cefoxitin (FOX) and vancomycin (VA) were used for the analysis of antibacterial susceptibility of *S. aureus* strains. The combined effect of CH and antibiotics was examined using plates with MHA plus one-fourth of the MIC of CH, which was considered as a sub-inhibitory concentration [54,55]. The test strips were placed onto an agar surface and gently pressed with the sterile forceps to ensure the contact. Plates were incubated at 35 °C for 20 h in aerobic conditions. The susceptibility testing of each antibiotic for each isolate and for the reference strains was performed in triplicates. After the incubation MIC values were read and the median values were calculated.

### 4.7. Statistical Analysis

To compare MICs and MICs changes across MRSA and MSSA U Mann-Whitney test was used and the Kruskal-Wallis test was used to compare MICs and MICs changes across MLS_B_ negative, kMLS_B_ and iMLS_B_ strains. The *post-hoc* analysis was carried out using Bonferroni correction. The results from the synergism assay were submitted to the Wilcoxon Signed-Rank Test. For all used tests *p* ≤ 0.05 was considered as statistically significant. The data was analyzed with the use of STATISTICA v 10.0 software (StatSoft, Krakow, Poland) and Windows 10.

## 5. Conclusions

Our data showed that MSSA and MRSA clinical strains are susceptible to CH. This antimicrobial effect of CH varied across analyzed strains, probably due to intra-species diversity. The addition of CH to MHA significantly increased erythromycin and clindamycin antimicrobial activity. The synergy of CH and antibiotics activity *in vitro* can indicate the potential role of CH as a factor capable to augment the antibacterial activity of selected antibiotics *in vivo.*

## Figures and Tables

**Table 1:** Profile of resistance to methicillin and MLS_B_ antibiotics for examined strains and MIC of CH against examined *S. aureus* strains.

Strain	Cefoxitin Diameter of the Inhibition Zone (mm)	Presence of *mecA*	Methicillin Resistance Profile	Erythromycin Diameter of the Inhibition Zone (mm)	Clindamycin Diameter of the Inhibition Zone (mm)	Mechanism of Resistance to MLS_B_ Antibiotics	CH MIC (µg/mL)
*S. aureus* ATCC 25923	35	−	MSSA	25	25	-	2048
*S. aureus* ATCC 43300	21	+	MRSA	0	0	kMLS_B_	1024
*S. aureus* ATCC 6538	31	+	MRSA *	30	30	-	2048
*S. aureus* 1	34	−	MSSA	25	25	-	1024
*S. aureus* 2	32	−	MSSA	23	25	-	1024
*S. aureus* 3	31	−	MSSA	0	25	iMLS_B_	2048
*S. aureus* 4	32	+	MRSA *	25	27	-	1024
*S. aureus* 5	13	+	MRSA	0	30	iMLS_B_	1024
*S. aureus* 6	31	−	MSSA	30	35	-	1024
*S. aureus* 7	32	+	MRSA *	35	33	-	1024
*S. aureus* 8	31	−	MSSA	30	35	-	1024
*S. aureus* 9	30	+	MRSA *	35	25	-	1024
*S. aureus* 10	31	−	MSSA	10	22	iMLS_B_	256
*S. aureus* 11	31	−	MSSA	21	22	-	1024
*S. aureus* 12	8	+	MRSA	0	0	kMLS_B_	1024
*S. aureus* 13	14	+	MRSA	0	0	kMLS_B_	1024
*S. aureus* 14	0	+	MRSA	0	0	kMLS_B_	2048
*S. aureus* 15	21	+	MRSA	25	30	-	1024
*S. aureus* 16	18	+	MRSA	0	0	kMLS_B_	1024
*S. aureus* 17	11	+	MRSA	0	0	kMLS_B_	1024
*S. aureus* 18	19	+	MRSA	25	30	-	256
*S. aureus* 19	14	+	MRSA	0	0	kMLS_B_	1024
*S. aureus* 20	19	+	MRSA	0	0	kMLS_B_	256

CH: catechin hydrate, MIC: minimal inhibitory concentration, MRSA: methicillin resistance *Staphylococcus aureus*, according to the EUCAST recommendations FOX ≤ 22 mm [44 MSSA: methicillin susceptible *Staphylococcus aureus*, according to the EUCAST recommendations FOX > 22 mm [44], MRSA *: phenotype and genotype discordance, kMLS_B_: constitutive macrolide, lincosamide and streptogramin B mechanism, according to the EUCAST recommendations E < 18 mm and DA < 19 mm [44] iMLS_B_: inducible macrolide, lincosamide and streptogramin B mechanism, according to the EUCAST recommendations E < 18 and DA ≥ 22 (D-shaped zone of inhibition around clindamycin with flattening towards erythromycin disc [44].

**Table 2:** The combined antibacterial effects of catechin hydrate and erythromycin, clindamycin, cefoxitin and vancomycin on *Staphylococcus aureus* examined strains (MIC values expressed in µg/mL).

Bacterial Strain	E	E + CH	∆%	DA	DA + CH	∆%	FOX	FOX+CH	∆%	VA	VA + CH	∆%
*S. aureus* ATCC 25923	0.38	0.50	32%	0.064	0.064	0%	1	1	0%	0.75	1	33%
*S. aureus* ATCC 43300	256	256	0%	256	256	0%	12	8	−33%	0.38	0.75	97%
*S. aureus* ATCC 6538	0.064	0.19	197%	0.023	0.032	39%	2	1.5	−25%	0.50	0.38	−24%
*S. aureus* 1	0.50	0.19	−62%	0.064	0.032	−50%	2	1.5	−25%	0.75	0.50	−33%
*S. aureus* 2	0.50	0.25	−50%	0.064	0.064	0%	0.75	1.5	100%	0.38	0.75	97%
*S. aureus* 3	256	256	0%	0.023	0.016	−30%	1.5	1.5	0%	0.50	0.50	0%
*S. aureus* 4	0.38	0.064	−83%	0.064	0.023	−64%	2	1.5	−25%	0.50	0.38	−24%
*S. aureus* 5	256	4	−98%	0.094	0.064	−32%	256	24	−91%	0.75	0.50	−33%
*S. aureus* 6	0.50	0.25	−50%	0.064	0.032	−50%	1.5	2	33%	0.38	0.38	0%
*S. aureus* 7	0.38	0.125	−67%	0.032	0.016	−50%	1	0.25	−75%	0.50	0.25	−50%
*S. aureus* 8	0.19	0.064	−66%	0.032	0.016	−50%	1.5	1	−33%	0.38	0.50	32%
*S. aureus* 9	0.38	0.19	−50%	0.064	0.016	−75%	1	2	100%	0.38	0.50	32%
*S. aureus* 10	32	32	0%	0.047	0.047	0%	2	2	0%	0.38	0.38	0%
*S. aureus* 11	0.38	0.094	−75%	0.047	0.023	−51%	1.5	2	33%	0.38	0.25	−34%
*S. aureus* 12	256	256	0%	256	256	0%	256	256	0%	0.75	0.50	−33%
*S. aureus* 13	256	256	0%	256	256	0%	32	32	0%	0.75	0.50	−33%
*S. aureus* 14	256	256	0%	256	256	0%	256	256	0%	0.75	0.50	−33%
*S. aureus* 15	0.25	0.38	52%	0.064	0.047	−27%	8	6	−25%	0.38	0.75	97%
*S. aureus* 16	256	256	0%	256	256	0%	256	256	0%	0.50	0.50	0%
*S. aureus* 17	256	256	0%	256	256	0%	256	2	−99%	0.38	0.38	0%
*S. aureus* 18	0.38	0.25	−34%	0.047	0.047	0%	6	1	−83%	0.50	0.38	−24%
*S. aureus* 19	256	256	0%	256	256	0%	256	256	0%	0.50	0.50	0%
*S. aureus* 20	256	256	0%	256	256	0%	12	4	−67%	0.38	0.38	0%
Median	0.5	0.5	0	0.064	0.047	0	2	2	0	0.50	0.5	0
Q1	0.38	0.19	0	0.047	0.023	0	1.5	1,5	0	0.38	0.38	−32
Q3	256	256	62	256	256	50	256	32	33	0.75	0.5	33
*p*		0.009			0.006			0.064			0.605	

CH: catechin hydrate, E: erythromycin, DA: clindamycin, FOX: cefoxitin, VA: vancomycin, Q1: lowest quartile, Q3: upper quartile.

## Abbreviations

The following abbreviations are used in this manuscript:

CFU: colony forming-unit
CH: catechin hydrate
DA: clindamycin
E: erythromycin
EUCAST: European Committee for Antimicrobial Susceptibility Testing
FOX: cefoxitin
iMLS_B_: inducible macrolide, lincosamide and streptogramin B mechanism
kMLS_B_: constitutive macrolide, lincosamide and streptogramin B mechanism
MHA: Mueller-Hinton agar
MIC: minimal inhibitory concentration
MRSA: methicillin resistance *Staphylococcus aureus*
MSSA: methicillin susceptible *Staphylococcus aureus*
VA: vancomycin
